# The efficacy of Yiqi Huoxue method in treating coronary artery disease after percutaneous coronary intervention: A meta-analysis in accordance with PRISMA guideline

**DOI:** 10.1097/MD.0000000000030739

**Published:** 2022-10-14

**Authors:** Miao Zhang, Ming-Yue Sun, Qi-Ting Chen, Feng-Qin Xu, Zong-Zheng Chen, Wen-Bo Wei, Rui-Ting Wang, Gui-Peng Xu, Hui-Jun Yin

**Affiliations:** a Shenzhen Second People’s Hospital, First Affiliated Hospital of Shenzhen University, Shenzhen, Guangdong Province, P.R. China; b Department of Cardiovascular Disease, Beijing Xiyuan Hospital, China Academy of Chinese Medical Sciences, Beijing, P.R. China; c Dongzhimen Hospital, Beijing University of Chinese Medicine, Beijing, P.R. China; d Graduate School, Gansu University of Chinese Medicine, Lanzhou, Gansu, P.R. China.

**Keywords:** coronary artery disease, meta-analysis, percutaneous coronary intervention, Yiqi Huoxue

## Abstract

Percutaneous coronary intervention (PCI), the most common method in treating coronary artery disease (CAD), has a variety of side effects. Yiqi Huoxue therapy (YQHX) can effectively alleviate the symptoms of patients and reduce the side effects. However, a reliable and systematic assessment of the methodologies is not available.

**Methods:**

Seven electronic databases were searched to identify randomized controlled trials of YQHX method for CAD after PCI. The quality assessment of the trials included was performed by employing the Cochrane Risk of Bias tool.

**Results:**

One thousand eight hundred sixty-eight patients from 23 randomized controlled trials were included in this review. The aggregated results showed that the experimental group got better effect in increasing ORR, TCMSRR, ECG, HDL-C, and in lowering the level of CRP, TC, and MACE in comparison with the control group.

**Conclusion:**

YQHX method is a valid complementary and alternative therapy in the management of CAD after PCI, and is an effective and safe therapy for CAD.

## 1. Introduction

Coronary artery disease (CAD) is one of the most serious cardiovascular diseases threatening human health. Yiqi Huoxue therapy (YQHX) means invigorating Qi and activating blood circulation, and has already been widely used to treat cardiovascular diseases.^[1–3]^ Presently, percutaneous coronary intervention (PCI) is the most common method in treating CAD and can reduce the mortality of CAD significantly.^[3]^ However, PCI cannot root out the underlying causes or pathological basis of coronary stenosis and may damage blood vessels and endothelial cells, thus inducing platelet aggregation and thrombosis.^[4]^ Furthermore, the patients may also be complicated with coronary embolism and thrombosis, myocardial ischemia-reperfusion injury, coronary microcirculation disorder, no reflux, in-stent restenosis and other pathological conditions.^[5]^ Routine dual antiplatelet therapy after PCI also has toxic and side effects.^[6]^ In addition, PCI is an exogenous injury and is normally costly. Therefore, alternative and complementary therapies in CAD after PCI treatment are becoming more and more imminent.

According to the basic theories of Traditional Chinese Medicine (TCM), CAD after PCI is equivalent to the term of “Xiong Bi.” The etiology and pathogenesis of CAD after PCI are related to Qi deficiency and blood stasis referring to the 5 elements in TCM. When Qi deficiency occurs in the organism, blood circulation will become, to a different degree, retarded, which may lead to a blockage of the heart vessels.^[7]^ Therefore, invigorating Qi and activating circulation to remove blood stasis, the term for which in Chinese Pinyin is “Yiqi Huoxue,” are important therapies for CAD after PCI.^[8]^ Clinical studies have proven that YQHX can effectively alleviate the symptoms of the patients and reduce the side effects of drugs.^[5]^ However, either the evidence of the effect of YQHX on CAD after PCI is so far insufficient or the current information available is not systematic. Therefore, a meta-analysis of clinical randomized controlled trials (RCTs) was conducted to evaluate the efficacy and safety of YQHX on patients with CAD after PCI.

## 2. Methods

### 2.1. Search strategy

The Cochrane Library, PubMed, EMBASE, the China National Knowledge Infrastructure database, the Chinese Biomedical Literature database, the VIP database and the Wanfang database were searched. The search terms used were (Chinese medicine OR herbs OR herbal formula OR Yiqi OR Huoxue OR Supplementing Qi OR activating blood circulation) AND (CAD) AND (PCI OR intervention therapy) AND (RCT). The last search was finished on December 31, 2021. No limit was placed on the language.

### 2.2. Study selection

Studies were selected according to the Cochrane Handbook for Systematic Reviews of Interventions.^[9]^ *Inclusion Criteria.* The studies were performed as RCTs; patients were diagnosed with CAD and received PCI; dual antiplatelet therapy plus other western medicine was permitted to be taken according to individual symptoms; YQHX formula composed of classic herbs (Astragalus or Salvia miltiorrhiza or Codonopsis pilosula or Ginseng with clear dose) with conventional western medicine (CWM) was used for the experimental group and CWM alone for the control group. The primary outcomes included overall response rate (ORR) and TCM syndrome response rate (TCMSRR) by referring to the evaluation criteria of Guidelines for clinical research on Chinese new herbal medicines (Table 1), electrocardiogram (ECG) improvement (ST segment rose more than 0.05 MV after treatment, in the main lead, the change of T wave became more than 25% shallow or T wave changed from flat to upright), and major adverse cardiovascular events (MACE); secondary outcomes included levels of C-reactive protein (CRP) and blood lipid index (total cholesterol [TC], TG, high-density lipoprotein cholesterol [HDL-C], LDL-C).

**Table 1:** Evaluation criteria on the efficacy of clinical symptoms and TCM syndromes recommended by GCRNDTCM

Classification	Detailed description
Markedly effective	Clinical symptoms and signs completely disappeared, or the score ratio of clinical symptoms/TCM syndromes reduction to 70% or more
Effective	Clinical symptoms and signs were significantly reduced, with clinical symptoms/TCM syndrome score ratio reduction to 30%, but less than 70%
Invalid	Clinical symptoms and signs were partially reduced, with clinical symptoms/TCM syndrome score ratio reduction less than 30%
Pejorative	The score ratio of clinical symptoms or TCM syndromes got worse

GCRNDTCM = guidelines of clinical research of new drugs of traditional Chinese medicine, TCM = traditional Chinese medicine.

*Exclusion Criteria*. The target population was incongruent with diagnostic criteria of CAD and received PCI; the main intervention was mixed with too many measures; the studies were allocated neither with appropriate control nor with randomization; the studies had data missed or duplicate publication.

### 2.3. Data abstraction

Two authors (Miao Z and Mingyue S) independently screened the titles and abstracts of the achieved citations from primary searching. Full text of the articles of potential interest was downloaded for further evaluation. Those meeting inclusion criteria were included in the final review. The discrepancies in the process of selection were resolved by the third author (Huijun Y).

### 2.4. Quality assessment

The methodological quality of trials was assessed independently by 2 authors (Miao Z and Mingyue S) referring to criteria from the Cochrane Handbook for Systematic Review of Interventions. The items included random sequence generation, allocation concealment, blinding, incomplete outcome data, selective outcome reporting, and other bias (defined as baseline data comparability).

### 2.5. Statistical method

Meta-analyses of RCTs were performed by using RevMan 5.3. Data were summarized by using risk ratio (RR) with 95% confidence intervals (CI) for discontinuous outcomes, or standard mean difference (SMD) with 95% CI for continuous outcomes. The data were assessed by both fixed effect model and random effect model. Publication bias was assessed by funnel plot analysis if the group included more than 10 trials.^[12]^

## 3. Results

### 3.1. Study selection

**Figure 1.**
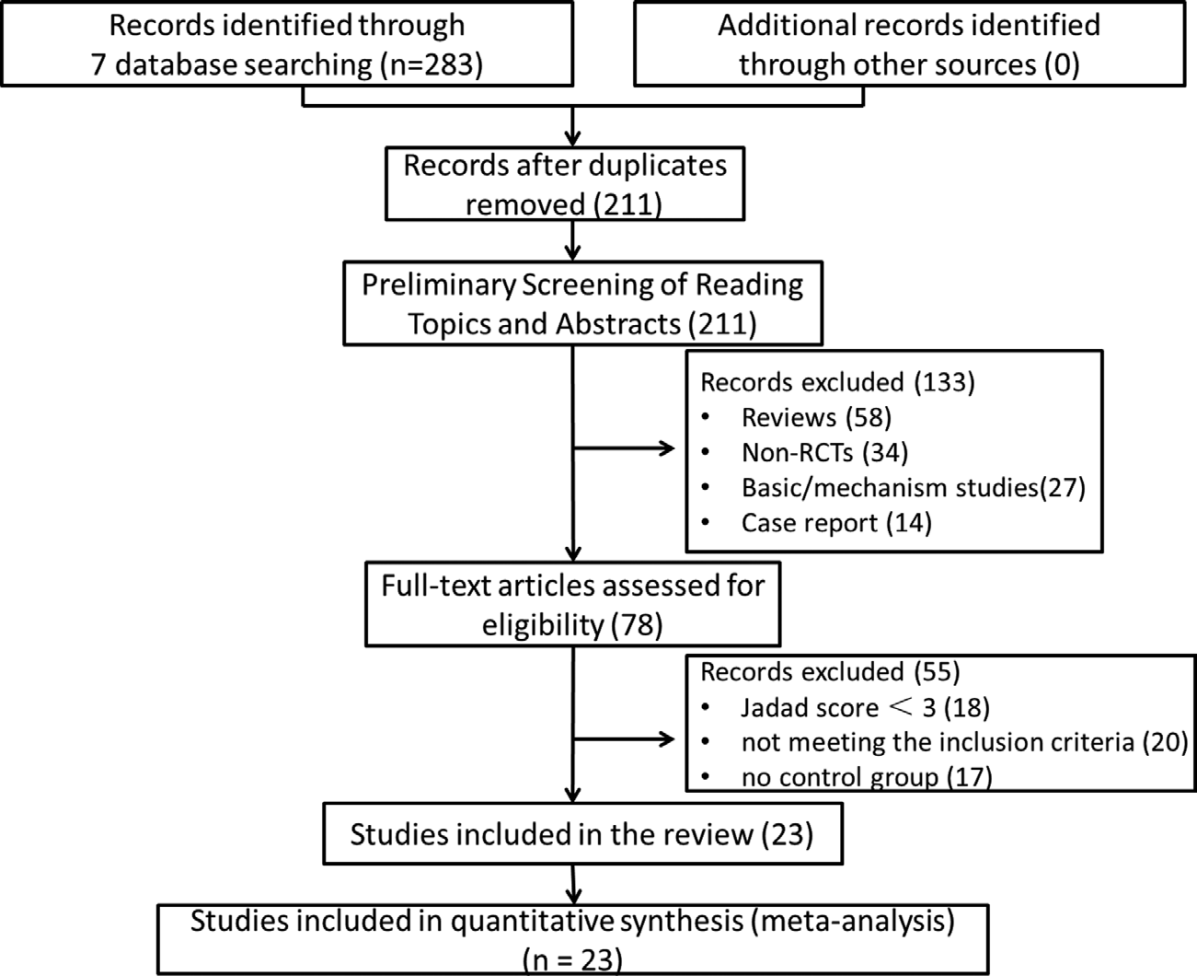
Flow diagram of study selection and identification.

The search of 7 databases (English or Chinese) identified 283 records for further evaluation (Fig. 1). 23 RCTs of them were eligible.^[4–26]^ All studies involved patient consent, and the informed consent was given.

### 3.2. Study characteristics

All of the 23 trials included were conducted in China and published in Chinese. All studies were performed in China, with a total of 1868 patients involved (936 the in control group, and 932 in the experimental group). In addition, all the studies exhibited comparable baseline patient characteristics, including age and gender (1053 male, and 815 female). The characteristics of the selected studies are shown in Table 2.

**Table 2:** Characteristics of included studies.

ID (Author/yr)	Case (T/C)	Age (T/C)	Gender (M/F)	Control group	Intervention group	Treatment duration	Outcome
Liu N/2016	30/30	NA	32/28	CWM	YQHX + CWM	14D	ORR,TCMSRR,ECG,CRP
Li YC/2017	30/30	65.23/62.14	31/29	CWM	YQHX + CWM	35D	TCMSRR,ECG
Ge YB/2015	38/38	64.68/62.05	45/31	CWM	YQHX + CWM	14D	TCMSRR,ECG
Wang Y/2015	30/30	63.78/60.54	31/29	CWM	YQHX + CWM	15D	ORR,TCMSRR,ECG,TC,TG,HDL-C,LDL-C
Dai GF/2010	49/46	61.2/63.8	54/41	CWM	YQHX + CWM	1Y	ORR,MACE,TCMSRR,ECG
Dai GF/2017	70/70	72.47/65.17	85/55	CWM	YQHX + CWM	6M	MACE,TCMSRR,ECG
Mao D/2016	30/30	55.3/56.2	34/26	CWM	YQHX + CWM	6M	MACE,ORR
Guo SF/2017	30/30	68.2 ± 4.5	40/20	CWM	YQHX + CWM	6M	TCMSRR,MACE,TC,TG
Yang JL/2017	46/45	60.18/61.3	50/41	CWM	YQHX + CWM	28D	ORR,TCMSRR,ECG,CRP,TC,TG,HDL-C,LDL-C
Guo ZY/2018	54/54	62.1/58.6	63/45	CWM	YQHX + CWM	56D	ORR
Zhai Y/2016	36/36	NA	34/38	CWM	YQHX + CWM	28D	ORR
Shi QJ/2014	30/30	60.3/62.8	38/22	CWM	YQHX + CWM	6M	MACE,ORR,ECG
Zhang Y/2011	30/30	64.2/65.6	28/32	CWM	YQHX + CWM	56D	ORR,ECG,CRP,TC,TG,HDL-C,LDL-C
Shi L/2017	34/34	65.3/64.7	42/26	CWM	YQHX + CWM	3M	TC,TG,HDL-C,LDL-C
Zhou CJ/2018	32/32	62.9/62.7	35/29	CWM	YQHX + CWM	6M	MACE,CRP
Zhang Y/2017	36/36	57.64/57.19	44/28	CWM	YQHX + CWM	6M	MACE,CRP
Zhou YL/2018	50/50	62.41/62.7	56/44	CWM	YQHX + CWM	6M	ORR,MACE
An HY/2017	30/30	63.43/63.70	33/27	CWM	YQHX + CWM	3M	ORR,TC,TG,HDL-C,LDL-C
Zhang LG/2019	36/36	48/9/48.2	39/33	CWM	YQHX + CWM	20D	MACE
Zhang W/2019	80/80	54.5/53.5	83/77	CWM	YQHX + CWM	6M	ORR
Huang BC/2019	30/30	63.58/63.42	33/27	CWM	YQHX + CWM	28D	CRP,TC,TG,HDL-C,LDL-C
Wu H/2020	53/53	63.37/63.76	58/48	CWM	YQHX + CWM	30D	ORR
Lin SM/2021	52/52	60.8/60.3	65/39	CWM	YQHX + CWM	56D	TC,TG,LDL-C

CRP = C-reactive protein, CWM = conventional western medicine, ECG = electrocardiogram, HDL-C = high-density lipoprotein cholesterol, LDL-C = low density lipoprotein-cholesterol, MACE = major adverse cardiovascular events, NA = not applicable, ORR = overall response rate, TC = total cholesterol, TCMSRR = TCM syndrome response rate, TG = triglycerides, YQHX = Yiqi Huoxue.

### 3.3. Study quality

**Figure 2.**
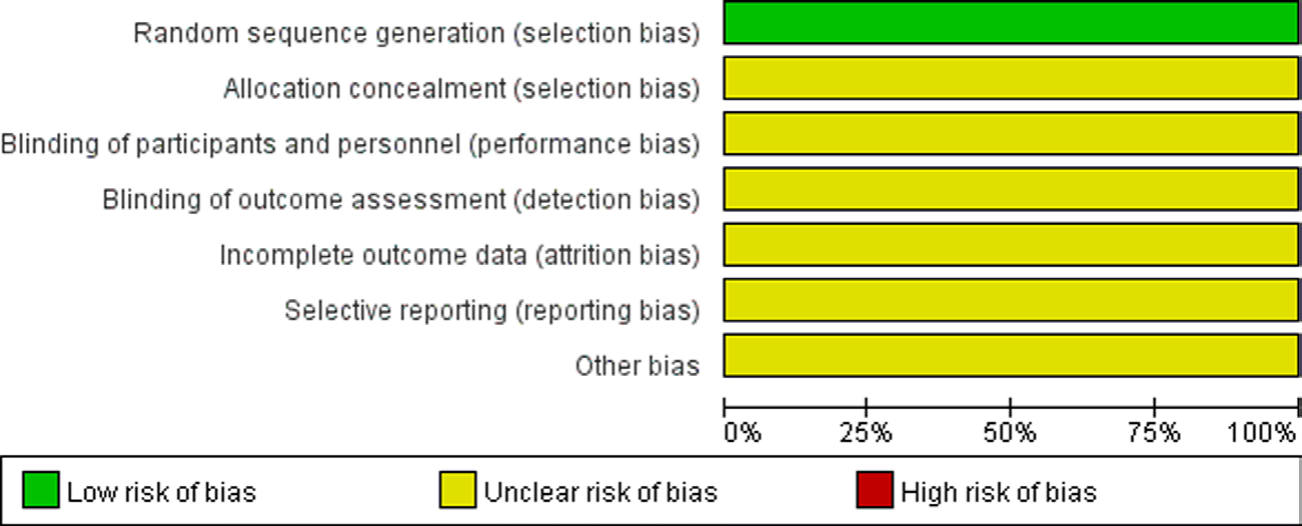
Risk of bias: reviewing authors’ judgments about each risk of bias item for each included study.

**Figure 3.**
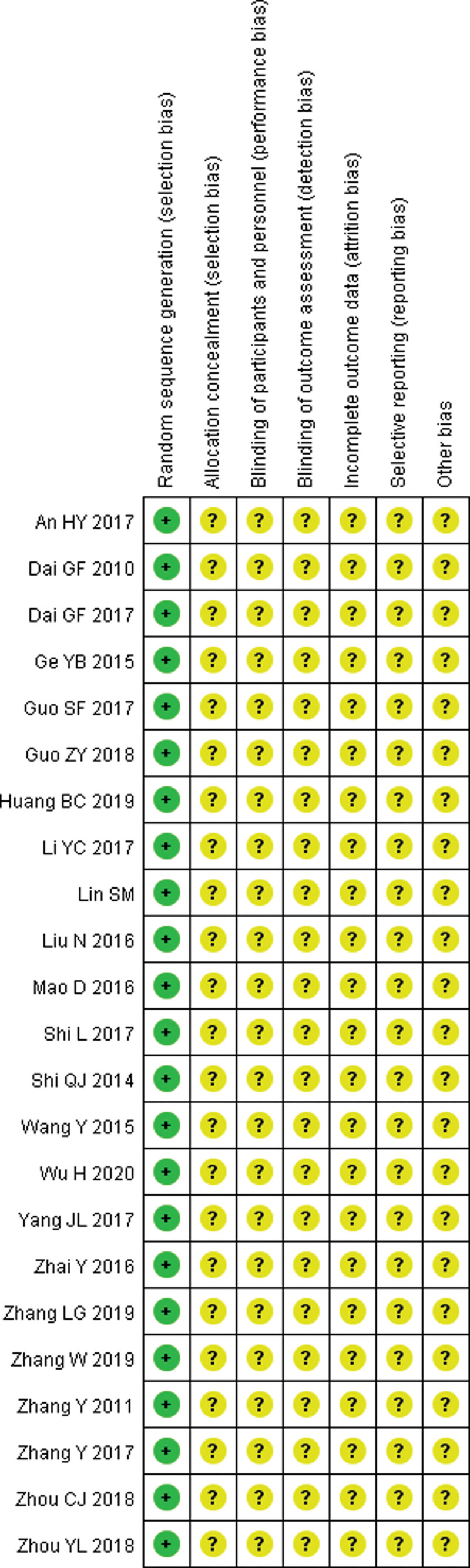
Summary of the risk of bias assessment for included trials.

Among the trials, 14 studies^[5–17,25]^ stated the method of the sequence generation with random number table and drawing, while none of the 22 studies reported details for sample size calculations and none was double-blind or placebo controlled study. Additionally, none mentioned allocation concealment or blinding methods. Fourteen trials included^[5–9,12–15,17–20,22]^ were assessed as low risk of bias in incomplete outcome data. Fourteen of the trials included^[5,6,8,10,11,13,14,16–22]^ were assessed as low risk of reporting bias, and the other 4,^[7,9,12,15]^ as unclear risk of reporting bias. The details of the risk of bias of each trial are presented in Figures 2 and 3.

### 3.4. Effects of the interventions

The outcomes, ORR (13 trials), TCMSRR (8 trials), ECG (9 trials), MACE (9 trials), blood lipid (TC, TG) level (7 trials), (HDL-C, LDL-C) level (6 trials), and CRP (6 trials) were analyzed.

#### 3.4.1. Overall response rate

**Figure 4.**
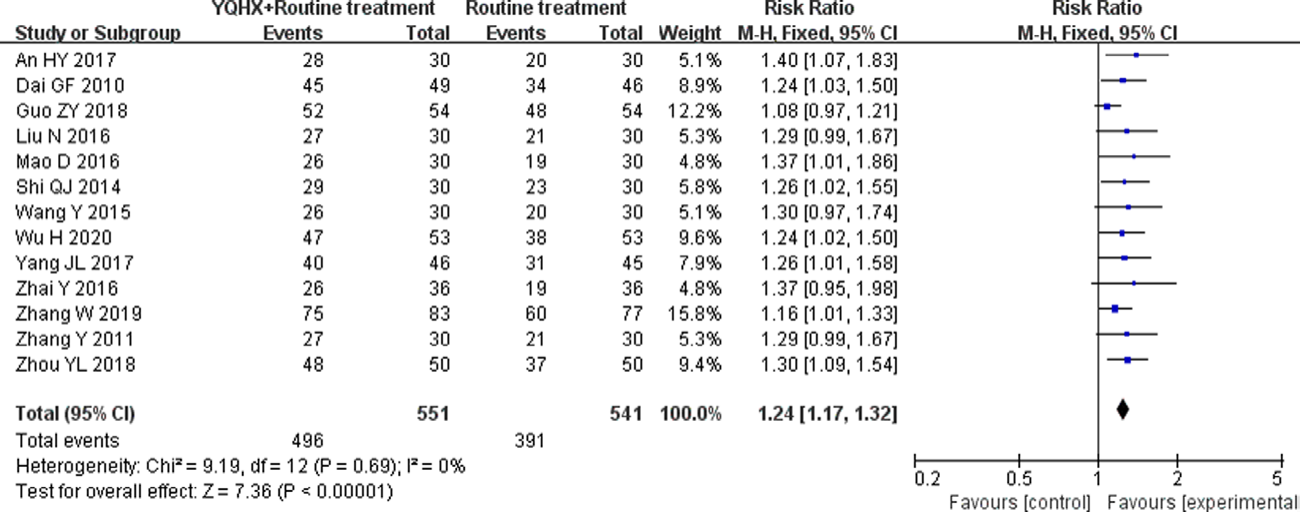
Forest plot of overall response rate.

 Thirteen RCTs^[5,6,8–10,12,14,15,17,19,21,23,25]^ reported ORR and found an obvious difference (RR = 1.24, 95% CI 1.17–1.32, 1092 participants, *P* < .00001), which meant that YQHX plus CWM was significantly better than CWM. No heterogeneity was found (*I*^2^ = 0%, *P* = .69) (Fig. 4).

#### 3.4.2. ECG improvement

**Figure 5.**
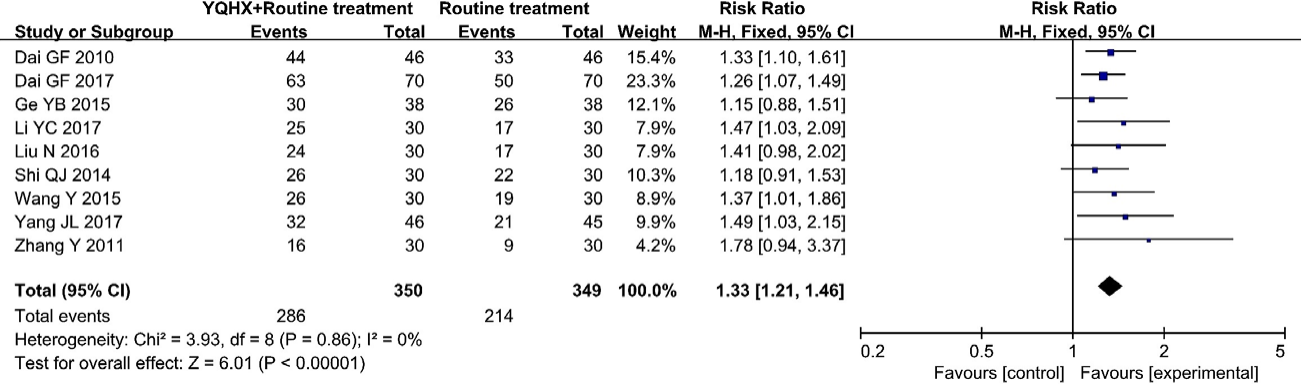
Forest plot of improvement of ECG. ECG = electrocardiogram.

Nine RCTs^[9,10,12,13,15,18,19,21,22]^ evaluated the effect of ECG improvement and found an significant difference (RR = 1.33, 95% CI 1.21–1.46, 699 participants, P < .0001). The result indicated that YQHX plus CWM was significantly better than CWM in ECG improvement, and there was significant homogeneity (*I*^2^ = 0%, *P* = .86) (Fig. 5).

#### 3.4.3. TCM syndrome response rate

**Figure 6.**
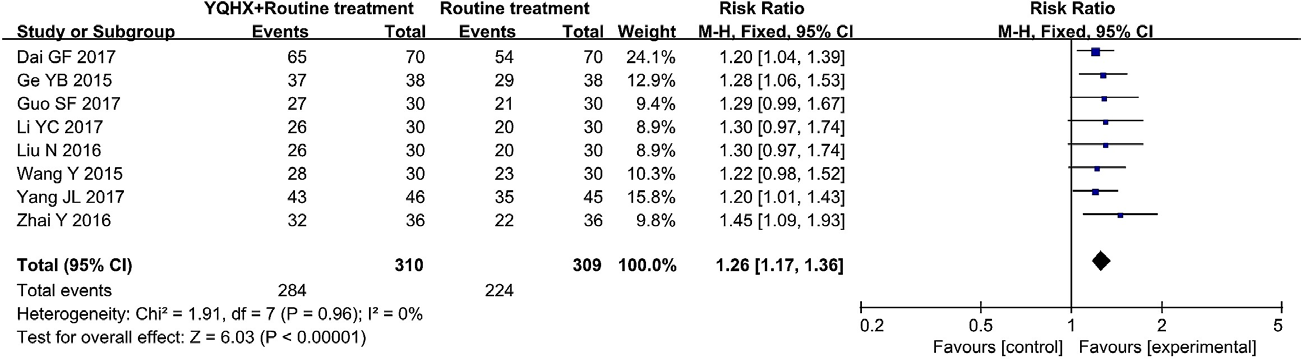
Forest plot of TCM syndrome response rate. TCM = traditional Chinese medicine.

Eight RCTs^[9,11–14,18,21,22]^ reported TCMSRR and found an obvious difference (RR = 1.26, 95% CI 1.17–1.36, 508 participants, *P* < .00001). The result indicated that YQHX combined with CWM was significantly better than CWM in the TCMSRR and there was significant homogeneity (*I*^2^ = 0%, *P *= .96) (Fig. 6).

#### 3.4.4. Major adverse cardiovascular events

**Figure 7.**
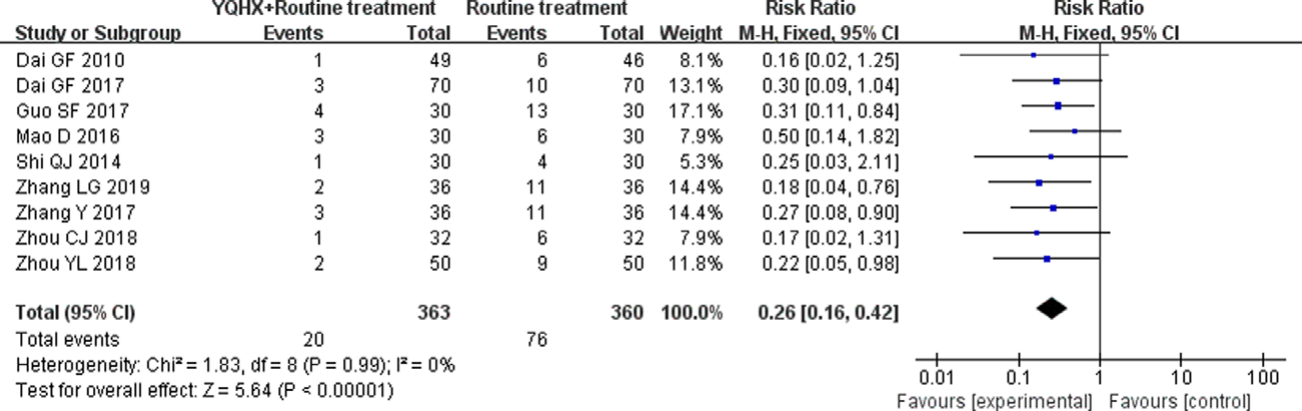
Forest plot of improvement of MACE. MACE = major adverse cardiovascular events.

Nine RCTs^[4,6,10,11,13,16,17,19,20]^ reported MACE and found an obvious difference (RR = 0.26, 95% CI 0.16–0.42, 723 participants, *P* < .00001). The result indicated that YQHX combined with CWM were significantly better than CWM in the MACE and there was significant homogeneity (*I*^2^ = 0%, *P* = .99) (Fig. 7).

#### 3.4.5. GRADE assessment

However, due to the poor methodology of the studies included and the obvious statistical heterogeneity among trials, quality of the evidence for all 4 outcomes (ORR, TCMSRR, ECG, MACE) were “low” and “very low,” according to the GRADE assessment (see Table 3).

**Table 3:** Summary of finding table of Yiqi Huoxue formula with conventional western medicine for patients were diagnosed with CAD and received PCI.

Patient: Patients were diagnosed with CAD and received PCI. Settings: Outpatient department/ Inpatient department. Intervention: Yiqihuoxue formula with conventional western medicine. Control: Conventional western medicine.						
Outcomes	Illustrative comparative risks* (95% CI)		Relative effect(95% CI)	No of participants(studies)	Quality of the evidence(GRADE)	Comments
	Assumed risk	Corresponding risk				
	**Control**	**Yiqi Huoxue**				
Overall response rate	**723 per 1000**	**904 per 1000**(846 to 955)	**RR 1.25** (1.17 to 1.32)	986(12 studies)	⊕⊕⊝⊝low*,†	Further research is very likely to have an important impact on our confidence in the estimate of effect and is likely to change the estimate.
TCM syndrome response rate	**725 per 1000**	**913 per 1000**(848 to 986)	**RR 1.26** (1.17 to 1.36)	619(8 studies)	⊕⊕⊝⊝low*,†	Further research is very likely to have an important impact on our confidence in the estimate of effect and is likely to change the estimate.
ECG improvement	**613 per 1000**	**816 per 1000**(742 to 895)	**RR 1.33** (1.21 to 1.46)	699(9 studies)	⊕⊕⊝⊝low*,†	Further research is very likely to have an important impact on our confidence in the estimate of effect and is likely to change the estimate.
Major adverse cardiovascular events	**211 per 1000**	**55 per 1000**(34 to 89)	**RR 0.26** (0.16 to 0.42)	723(9 studies)	⊕⊕⊝⊝low*,†	Further research is very likely to have an important impact on our confidence in the estimate of effect and is likely to change the estimate.

* There were serious limitations of methodological quality of included trials according to the risk of bias assessment.

† There were serious publication bias between the studies.

CAD = coronary artery disease, CI = confidence interval, ECG = electrocardiogram, PCI = percutaneous coronary intervention, RR = risk ratio, TCM = Traditional Chinese Medicine.

#### 3.4.6. CRP

**Figure 8.**
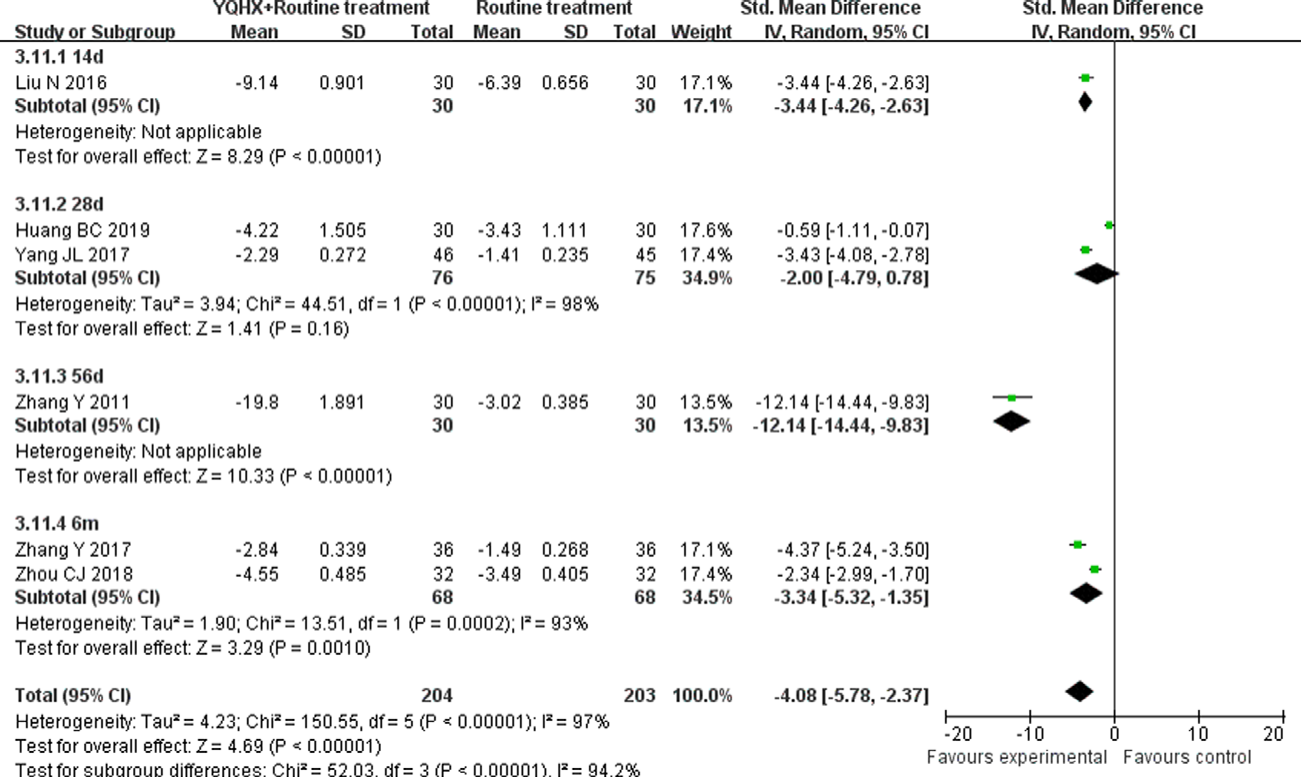
Forest plot of improvement of CRP. CRP = C-reactive protein.

Six RCTs^[9,12,15–17,24]^ reported CRP and found an obvious difference (SMD = −4.08, 95% CI − 5.78–2.37, 407 participants, *P* < .00001). The trials were divided into 4 subgroups which were 14d, 28d, 56d and 6m by course of intervention. The meta-analysis of 3 subgroups of CRP assessment showed obvious differences (28d: SMD = -2.00, 95% CI -4.79 to 0.78, 151 participants, *P <* .00001; 6m: SMD = -3.34, 95% CI -5.32 to -1.35, 136 participants, *P *= .001) with significant heterogeneity (28d: *I*^2^ = 98%, *P* = .16; 6m: *I*^2^ = 93%, *P* < .00001). There was significant difference between subgroups (*I*^2^ = 94.2%, *P* < .00001) (Fig. 8).

#### 3.4.7. Blood lipid

**Figure 9.**
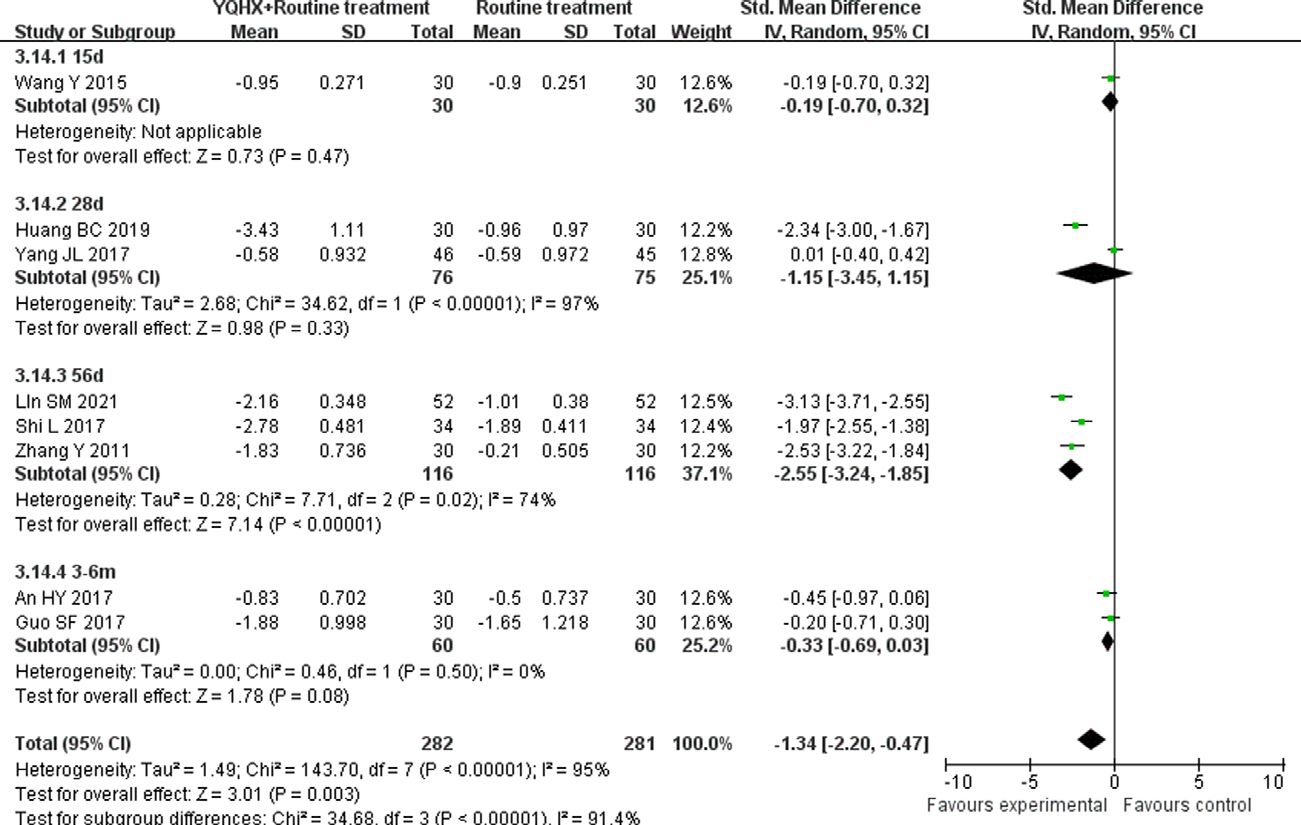
Forest plot of improvement of TC. TC = total cholesterol.

**Figure 10.**
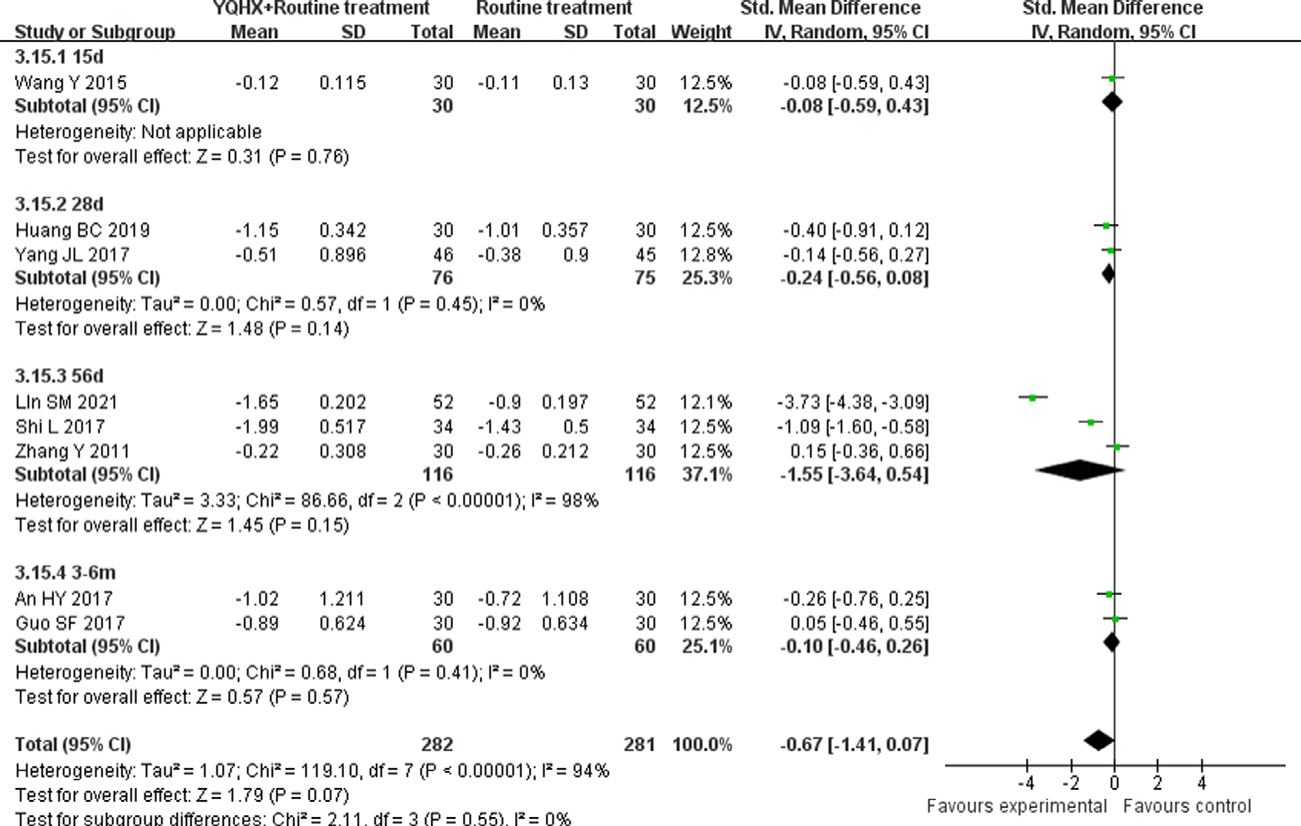
Forest plot of improvement of TG. TG = triglycerides.

**Figure 11.**
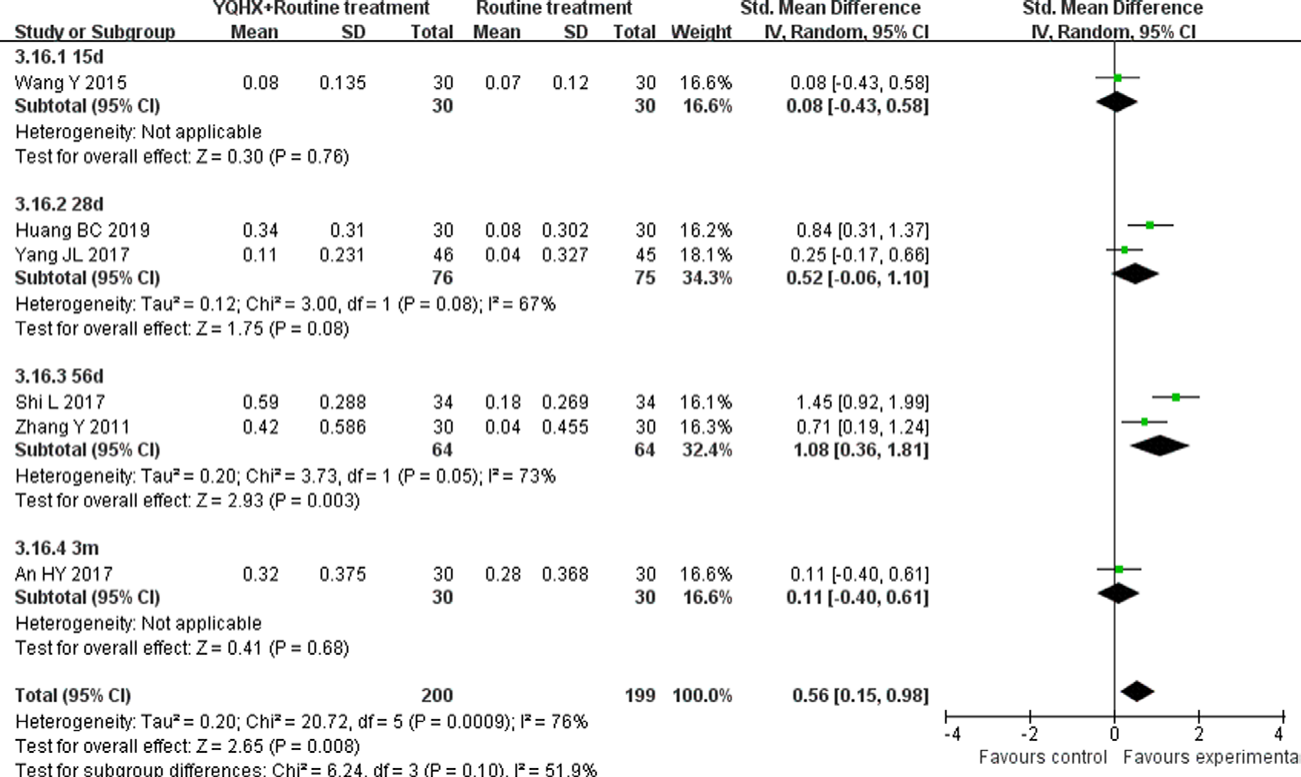
Forest plot of improvement of HDL-C. HDL-C = high-density lipoprotein cholesterol.

**Figure 12.**
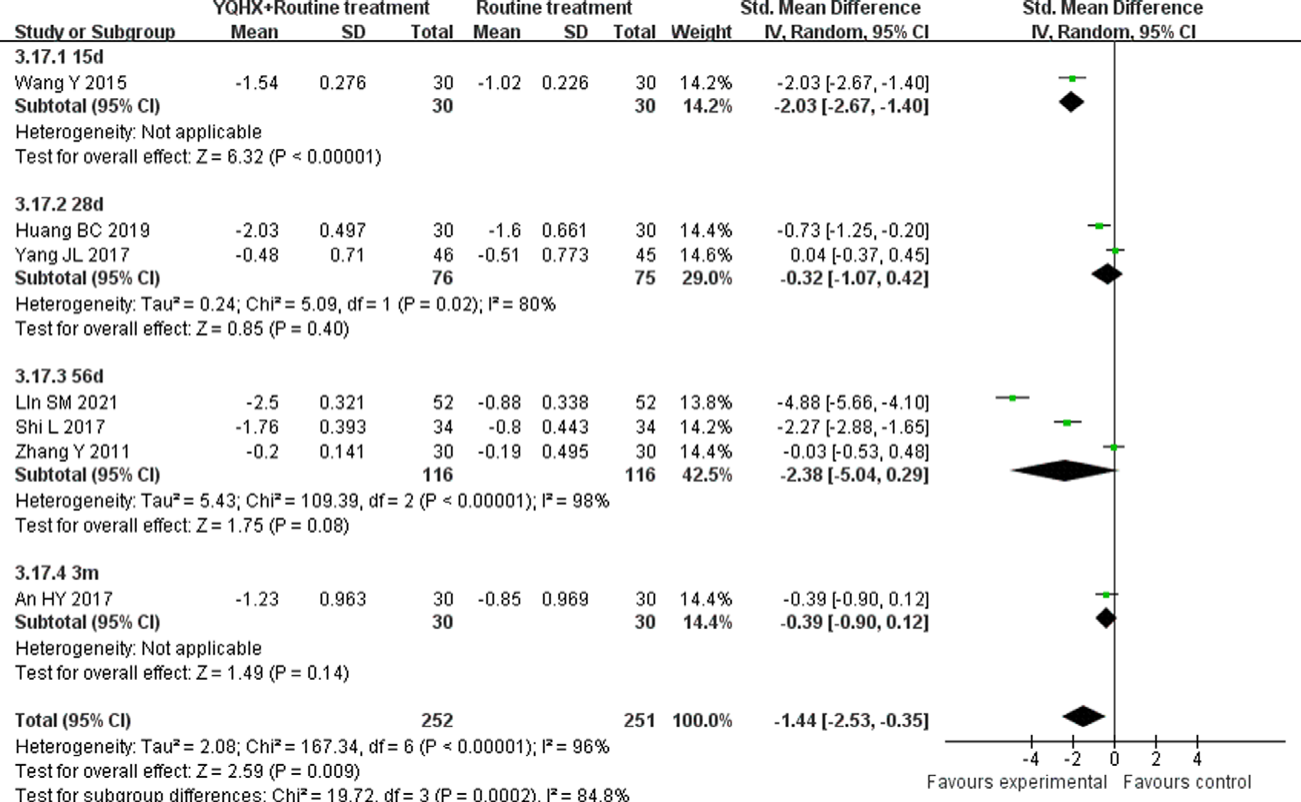
Forest plot of improvement of LDL-C. LDL-C = Low density lipoprotein-cholesterol.

**(1) *TC*** 8 RCTs^[5,7,11,12,15,21,24,26]^ reported TC and found an obvious difference (SMD = −1.34, 95% CI − 2.20 to − 0.47, 563 participants, *P* = .003). The trials were divided into 4 subgroups which were 15d, 28d, 56d, and 3-6m. The meta-analysis of 3 subgroups showed no significant differences (15d: SMD = -0.19, 95% CI -0.70 to 0.32, 60 participants, *P* = .47; 28d: SMD = -1.15, 95% CI -3.45 to 1.15, 151 participants, *P* = .33; 3-6m: SMD = -0.33, 95% CI -0.69 to 0.03, 120 participants, *P* = .08). In contrast, the 56d subgroup^[21,24]^ showed that 2 groups had obvious difference (SMD = -2.55, 95% CI -3.24 to -1.85, 232 participants, *P* < .00001) with low heterogeneity (*I*^2^ = 34%, *P* = .22). There was significant difference between subgroups (*I*^2^ = 91.9%, *P* < .00001) (Fig. 9).
**(2) *TG*** 8 RCTs^[5,7,11,12,15,21,24,26]^ reported TG and found no obvious difference (SMD = −0.67, 95% CI − 1.41 to 0.07, 563 participants, *P* = .07). In TG group, the trials were divided into 4 subgroups which were 15d, 28d, 56d and 3-6m. The meta-analysis of subgroups showed no significant differences (15d: SMD = -0.08, 95% CI -0.59 to 0.43, 60 participants, *P* = .76; 28d: SMD = -0.24, 95% CI -0.56 to 0.08, 151 participants, *P* = .14; 56d: SMD = -1.55, 95% CI -1.41 to 0.07, 232 participants, *P* = .15; 3-6m: SMD = -0.10, 95% CI -0.46 to 0.26, 120 participants, *P* = .57). There was no significant difference between subgroups (*I*^2^ = 0%, *P* = .55) (Fig. 10).
**(3) *HDL-C*** 6 RCTs^[5,11,12,15,21,24]^ reported HDL-C and found an significant difference (SMD = 0.56, 95% CI 0.15–0.98, 399 participants, *P* = .008). The trials were divided into 4 subgroups which were 15d, 28d, 56d and 3m. The meta-analysis of 3 subgroups showed no significant differences (15d: SMD = 0.08, 95% CI -0.43 to 0.58, 60 participants, *P* = .76; 28d: SMD = 0.52, 95% CI -0.06 to 0.10, 151 participants, *P* = .08; 3m: SMD = 0.11, 95% CI -0.40 to 0.61, 60 participants, *P* = .68). The 56d subgroup^[21,24]^ showed that 2 groups had obvious difference (SMD = 1.08, 95% CI 0.36 to 1.81, 128 participants, *P* < .05) with heterogeneity (*I*^2^ = 73%, *P* = .05). There was significant difference between subgroups (*I*^2^ = 51.9%, *P* = .10) (Fig. 11).
**(4) *LDL-C*** 7 RCTs^[5,11,12,15,21,24,26]^ reported LDL-C and found an obvious difference (SMD = −1.44, 95% CI -2.53 to -0.35, 503 participants, *P* = .0009). The trials were divided into 4 subgroups which were 15d, 28d, 56d and 3m. The meta-analysis of 3 subgroups showed no significant differences (28d: SMD = -0.32, 95% CI -1.07 to 0.42, 151 participants, *P* = .40; 56d: SMD = -2.38, 95% CI -5.04 to 0.28, 232 participants, *P* = .08; 3m: SMD = -0.39, 95% CI -0.90 to 0.12, 60 participants, *P* = .14). The 15d group^[14]^ showed that 2 groups had obvious difference (SMD = -2.03, 95% CI -2.67 to -1.40, 60 participants, *P* < .00001). There was significant difference between subgroups (*I*^2^ = 84.8%, *P* = .0002) (Fig. 12).

### 3.5. Adverse reactions

Five cases in the treatment group and 9 cases in the control group had adverse reactions, with an incidence of 4.1%, 7.5%, respectively. The incidence of adverse reactions in the treatment of PCI patients with YQHX was relatively lower.

### 3.6. Publication bias

**Figure 13.**
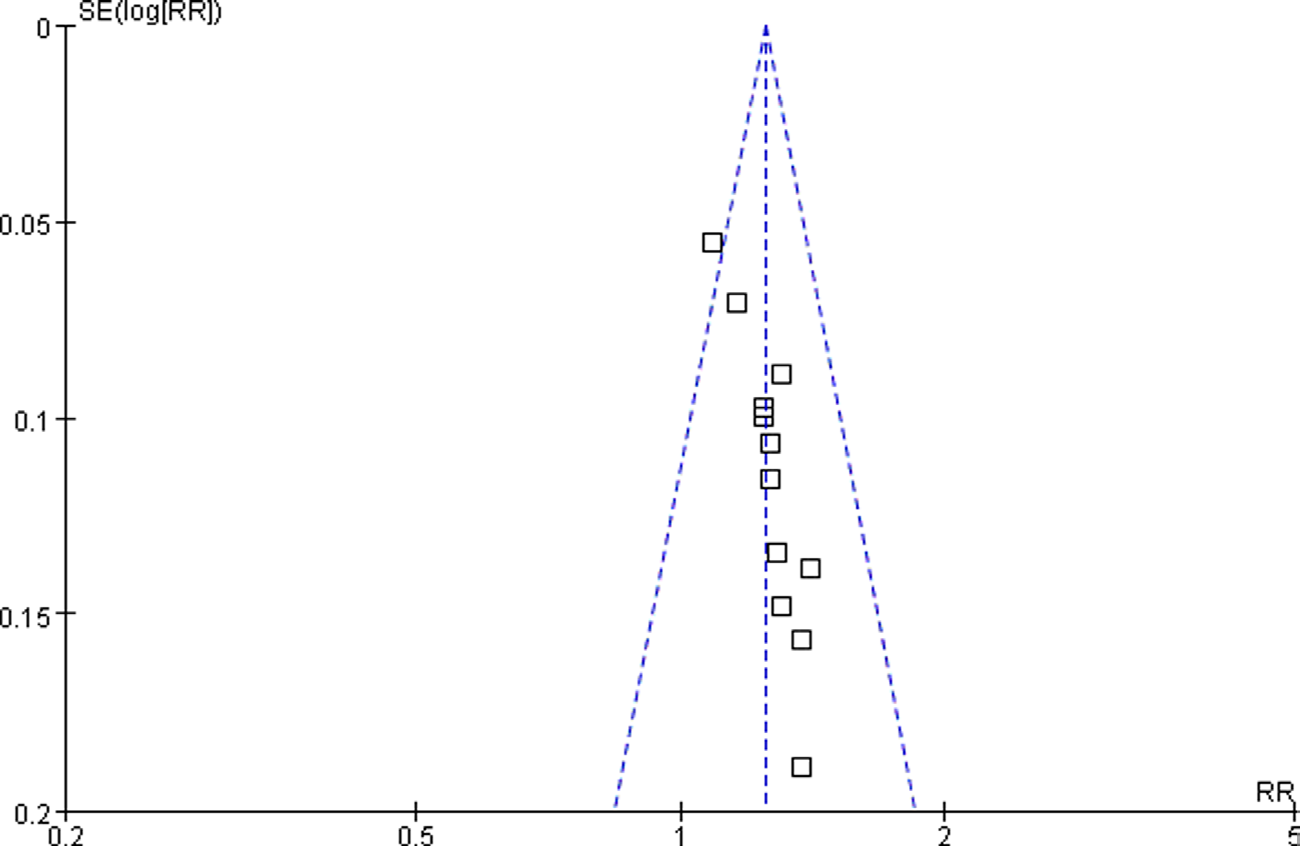
Funnel plot of the overall response rate.

In order to detect possible publication bias, the 13 trials of ORR were analyzed with a fixed effects model. The funnel plot of ORR was asymmetrical, indicating the presence of publication bias (Fig. 13).

## 4. Discussion

In this study, we systematically evaluated the RCT of the control group treated with CWM, while the experimental group were added YQHX. The ORR, ECG, TCMSRR, and MACE were the important indicators of clinical efficacy and quality of life of the patients.^[27,28]^ The results showed that these 4 outcomes of CAD patients after PCI treated with CWM alone were inferior to CWM plus YQHX herbs, which had higher safety and fewer side effects.

Inflammatory response is an important factor affecting plaque progression.^[35]^ CRP is a kind of protein secreted in the blood during acute inflammation and reflects the inflammatory status of the organism.^[29]^ The concentration of CRP in serum is positively correlated with the formation of atherosclerosis and the severity of CAD.^[30,31]^ Therefore, CRP can be used as an important index to measure the risk of CAD after PCI.^[37]^ The results showed that the combination of YQHX with CWM was better than CWM alone in reducing CRP.

Hyperlipidemia is one of the risk factors of atherosclerosis.^[32]^ Elevated blood lipids can damage endothelial cells, increase vascular permeability, cause cholesterol deposition, plaque formation, and thrombosis, and lead to coronary stenosis.^[33,34]^ Therefore, abnormal blood lipid level is an independent risk factor for restenosis after PCI. The results indicated that the combination of YQHX with CWM was better than CWM alone in reducing TC and increasing HDL-C.

PCI is currently recognized as the most effective and safe way to restore myocardial reperfusion when it meets the indications. PCI is an exogenous injury, and the persistent blood stasis is not relieved by the operation. The mechanical injury of PCI can also induce the formation of new blood stasis, then damage the local tissue, leading to the aggravation of blood stasis.^[35,36]^ PCI, having the function of “breaking blood,” is easy to consume the healthy Qi of the injured organism. The etiology and pathogenesis of PCI after CAD are Qi deficiency and blood stasis, for which YQHX is the basic therapy. Results from previous studies showed that YQHX, as a complementary treatment, may alleviate the clinical symptoms, reduce the onset of angina pectoris and the side effects of drugs, increase the exercise endurance, prevent in-stent restenosis, and improve the quality of life of CAD after PCI patients.^[28,37]^

Based on the above results, it can be concluded that the combination of YQHX with CWM is superior to CWM alone in the treatment of CAD after PCI. This integrated therapy has fewer adverse reactions and higher safety. However, the quality of the methodologies being used is not high enough. As all the studies were conducted in Chinese population, the results cannot necessarily be extrapolated to other populations. Therefore, more high-quality, multi-center, and largely sampled RCTs need to be carried out to provide more reliable evidence-based medical basis for clinical guidance.

## Acknowledgments

Thank all the authors for their contributions to this article.

## Author contributions

**Data curation:** Miao Zhang, Qiting Chen.

**Formal analysis:** Ming-Yue Sun, Qiting Chen.

**Funding acquisition:** Miao Zhang, Feng-Qin Xu.

**Investigation:** Hui-Jun Yin.

**Methodology:** Qiting Chen.

**Project administration:** Wenbo Wei.

**Software:** Miao Zhang, Qiting Chen.

**Supervision:** Zongzheng Chen, Hui-Jun Yin.

**Validation:** Zongzheng Chen.

**Visualization:** Ruiting Wang.

**Writing – original draft:** Miao Zhang, Ming-Yue Sun.

**Writing – review & editing:** Miao Zhang, Wenbo Wei, Feng-Qin Xu, Hui-Jun Yin.
